# Evaluation of an emergency medicine point-of-care ultrasound curriculum adapted for a resource-limited setting in Guyana

**DOI:** 10.1186/s12245-023-00531-y

**Published:** 2023-09-06

**Authors:** Rayal Jhagru, Rajiv Singh, Jordan Rupp

**Affiliations:** 1 Georgetown Public Hospital Corporation, Georgetown, Guyana; 2 Vanderbilt University Medical Center, Nashville, TN 37232 USA

**Keywords:** Ultrasound, Point-of-care ultrasound, Curriculum, Adapted curriculum, Global health, Competency assessment

## Abstract

**Background:**

This is a cohort pilot study of senior emergency medicine residents and residency-trained emergency medicine registrars practicing in an urban tertiary academic hospital in Guyana in South America. The primary aim was to assess the effectiveness of the current adapted residency ultrasound training curriculum and guide future ultrasound-specific continuing medical education. Ultrasound image acquisition, interpretation, and integration of ultrasound findings into clinical medical decision-making competency were assessed in a multimodal fashion: a written assessment and a practical assessment.

**Methods:**

This was a cross-sectional cohort pilot study of senior emergency medicine residents and registrars (residency graduates) practicing in Guyana, a low-middle-income country. Ultrasound image acquisition and interpretation competency were assessed in a multimodal fashion: a written assessment and a practical assessment. The results will be reported as simple percentages. Participants will be deemed competent if the combined score is greater than 80% on the assessment.

**Results:**

All senior residents and graduates of the residency program were competent in the core point-of-care ultrasound applications. The senior residents averaged 89% proficiency, and the residency graduates subdivided based on years since graduation averaged 87–100% proficiency. The more experienced providers performed better on the practical portion of the evaluation. Evaluating the composite analysis of all the participants revealed the Extended Focused Assessment with Sonography in Trauma (EFAST) exam (96%) and cardiac exam (93%) were done with the most proficiency.

**Conclusion:**

The assessment results of this pilot study suggest that the current residency ultrasound training curriculum adapted to the resources available is associated with sustained competency after graduation. There was minor attrition of knowledge amongst the senior physicians, but all senior residents and all participating residency graduates were competent in the core ultrasound applications.

## Introduction

Emergency medicine point-of-care ultrasound (POCUS) is the medical use of ultrasound technology for the bedside evaluation of emergency medical conditions and diagnoses [1]. POCUS performed and interpreted by emergency physicians is a fundamental skill in emergency medicine [1]. POCUS has established indications in assessment for free intraperitoneal fluid in trauma and identification of pregnancy location as examples. Point-of-care ultrasound use in the emergency department (ED) is associated with improved patient outcomes and increased patient satisfaction [2]. Physicians can become proficient in POCUS with modest training, and its use has been shown to improve diagnostic accuracy, reduce procedural complications, decrease inpatient length of stay, and improve patient satisfaction [3]. Ultrasound has been identified as a core skill required for graduating EM residents in the USA [2]. Emergency medicine has only recently been introduced in Guyana, a small English-speaking country in South America. As part of the development of emergency medicine in Guyana over the last 14 years, a POCUS training curriculum was introduced into the residency training program at Georgetown Public Hospital Corporation (GPHC) [4]. The curriculum was adapted to account for the limited resources available at the hospital. There are many challenges introducing a curriculum, especially if it is a short program [5]. The curriculum utilizes superuser direct observation of resident image acquisition and interpretation, structured observed ultrasound examinations, and minimum numbers of each of the core applications to achieve competency for graduation [4]. This curriculum is especially helpful where wireless Internet connectivity or image archival middleware is not available. POCUS has a wide range of application in the ED. It is incredibly important for diagnostic accuracy. Therefore, it is important to evaluate the competence of the physicians that most frequently use POCUS.

Competency is the ability of healthcare professionals to integrate knowledge, skills, values, and attitudes into individual patient care [6]. Applied to POCUS, competency requires combining medical knowledge, ability to acquire appropriate images, and cognitive aptitude to employ clinical ultrasound for optimized patient care and clinical outcomes [7]. Determining competency in POCUS is difficult. Methods of determining competency include traditional testing, testing using simulator models, video review, observation of bedside skills, over-reading of images by experienced sonologists, and monitoring of error rates through a quality assurance process [1]. There are several published efforts to assess and ensure competency during residency [8–12]. There is some data to show attrition of POCUS skills after residency. Kimura et al. retested thirty internal medicine physicians, deemed proficient at graduation and having passed a practical examination that confirms imaging skills and knowledge base when a score of ≥ 80% correct is attained [13]. None of the physicians (13) who had finished their residencies 2 or more years prior achieved the 80% pass mark. They concluded that learned skills in cardiac ultrasound diminish notably within 2 years of nonuse [13].

Resources are limited at GPHC leading to increased dependence on POCUS for patient care. There is limited access to cross-sectional imaging. While POCUS is currently a measured competency integrated into the residency program, that has not always been the case. Initial graduates received ultrasound training but did not complete an organized ultrasound curriculum. The ultrasound curriculum is designed to assure competency of residency graduates at the time of graduation. But the effectiveness of the training for persistent ultrasound competency has not previously been assessed. We aim to assess POCUS competency in senior emergency medicine residents and residency graduates of the Masters in Emergency Medicine Program at Georgetown Public Hospital Corporation to better inform residency training curriculum and improve ultrasound-specific continuing education efforts.

## Materials and methods

### Study setting and population

This was a cross-sectional cohort pilot study of senior emergency medicine residents (2nd and 3rd years) and residency-trained emergency medicine registrars practicing in an urban, tertiary, academic hospital in Guyana. The annual patient volume in the Accident & Emergency Department at GPHC is 80,000, both pediatric and adult patients. The emergency medicine residency is a 3-year program. The training structured training program was formally instituted in 2016. Given the lack infrastructure available to utilize image archival software, ultrasound training is centered around a minimum number of ultrasound scans, supervised scans, and directly observed ultrasound assessments. The number of required scans was based upon the available literature for each application. Additionally, residents are required to have a portion of each of their scans supervised by ultrasound trained faculty. The details are listed in Table 1 [4].

**Table 1:** GPHC residency ultrasound requirements for graduation

Application	Required no. of scans	Observed by EUS expert	Observed structured assessments
E-FAST	50	10	1st/3rd year
Cardiac	50	10	1st/3rd year
Renal	30	8	
Biliary	25	8	3rd year
Aorta	25	8	1st year
Pregnancy	30	10	1st year
Pulmonary	25	5	1st year
Other (misc.) applications	15		
Total	250		

The GPHC residency graduated its first class in 2013. Due to its size, there have been 18 total graduates of the program total. Two graduates have returned to their home islands to practice emergency medicine. One graduate works predominately in an administrative role and did not participate. Three graduates have fellowship training in ultrasound and were proctoring the assessments. Therefore, they were excluded from participating in the study. All senior residents participated in the assessment. Residency graduates ranged from one 1 to 10 years of practice experience post-graduation. The physicians participated on a volunteer basis and received no compensation. The evaluations occurred between June and July of 2022.

### Study protocol

Ultrasound image acquisition and interpretation competency were assessed in a multimodal fashion: a written assessment and a practical assessment. A written assessment was performed in two separate parts: the first part comprised of images or videos on a Microsoft PowerPoint presentation. The participant was required to interpret the images in a clinical context in short answers to the questions. The second part comprised of multiple-choice questions. The written portion assessed the physicians’ understanding of indications for POCUS testing, ability to interpret ultrasound images, and integration of ultrasound findings into clinical medical decision-making. The assessments were based on four core skills of POCUS (cardiac, EFAST, biliary, and pelvic). There is not a universally accepted written assessment tool available. The questions for the written assessment were formulated for this study. A pilot was done with the general medical officers (GMOs) in the ED to verify clarity and comprehensibility of the questions. The participant had 30 min to complete the written assessment. The practical assessment evaluates the physicians’ image acquisition and interpretation. It was done using the Ultrasound Competency Assessment Tool (UCAT) [11]. The subjects for the practical assessment were medical students, GMOs, or junior residents of the ED. The subjects were oriented to the study and given the option to quit at any time. Informed consent was taken from all of the subjects. The participant had 30 min to complete this phase of the assessment.

### Data analysis

The entire assessment was administered by the primary researcher. The results were stored on a secure password-protected flash drive and kept by the primary researcher. The data was tabulated in Microsoft Excel (Redmond, WA, USA). The results will be reported as simple percentages. Participants will be deemed competent if the combined score is greater than 80% on the assessment. Subgroup analysis will report performance by individual groups and performance based on ultrasound application.

## Results

Sixteen physicians of various experience levels were evaluated on competency of POCUS. There were 6 residents and 10 graduates. This represents 88.9% of the potential emergency physicians in Guyana that meet the inclusion criteria for this study.

There were three junior residents, those in their postgraduate year number 2 in training. They had a composite average score of 72%. The junior residents scored an average of 67% on the theory portion of the evaluation and 77% on the practical portion. The three senior residents in their final year of training had a composite average score of 89%. They scored 88% on the theory portion and 90% on the practical portion.

**Fig. 1:**
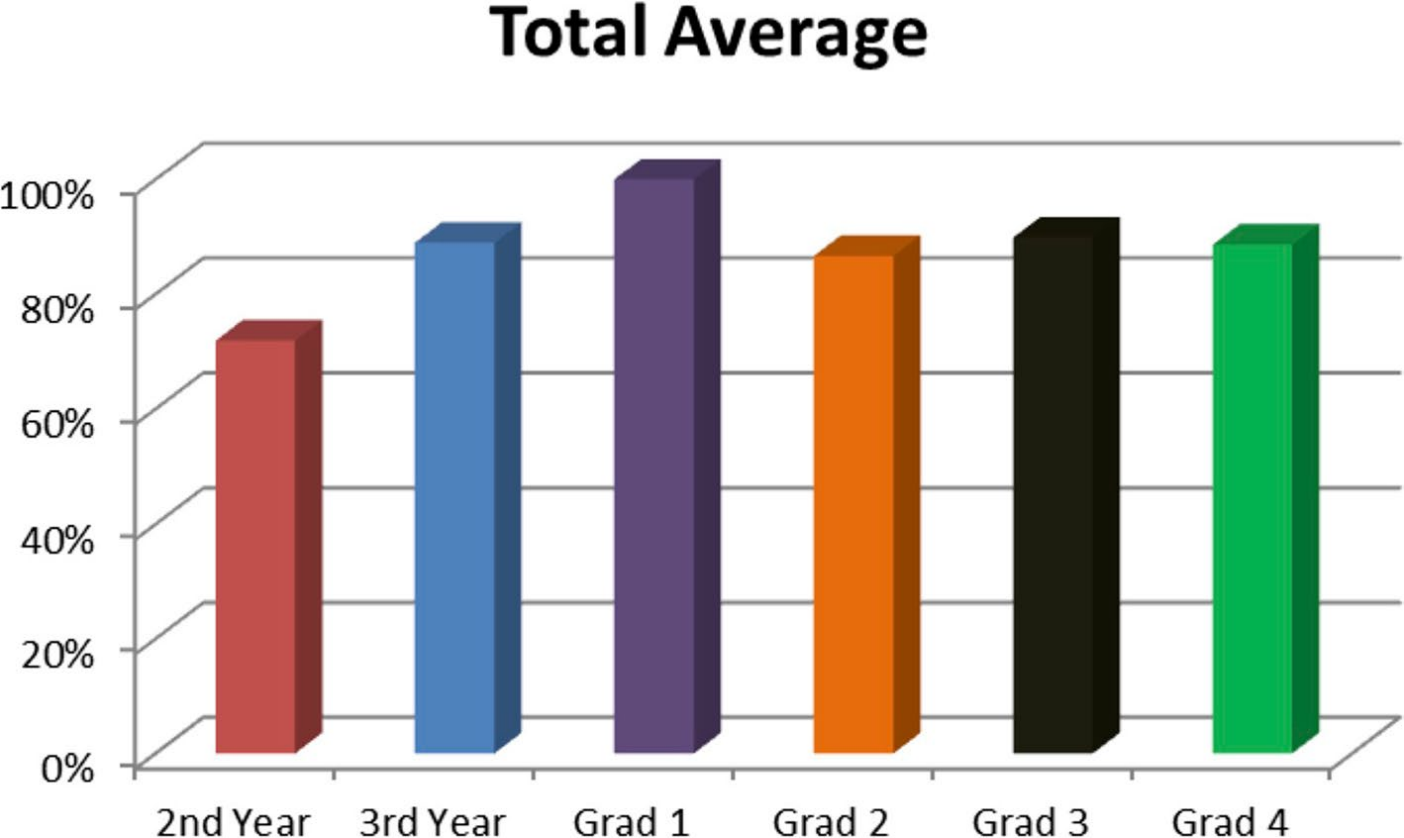
The average scores of the different levels of physicians evaluated based on experience

**Fig. 2:**
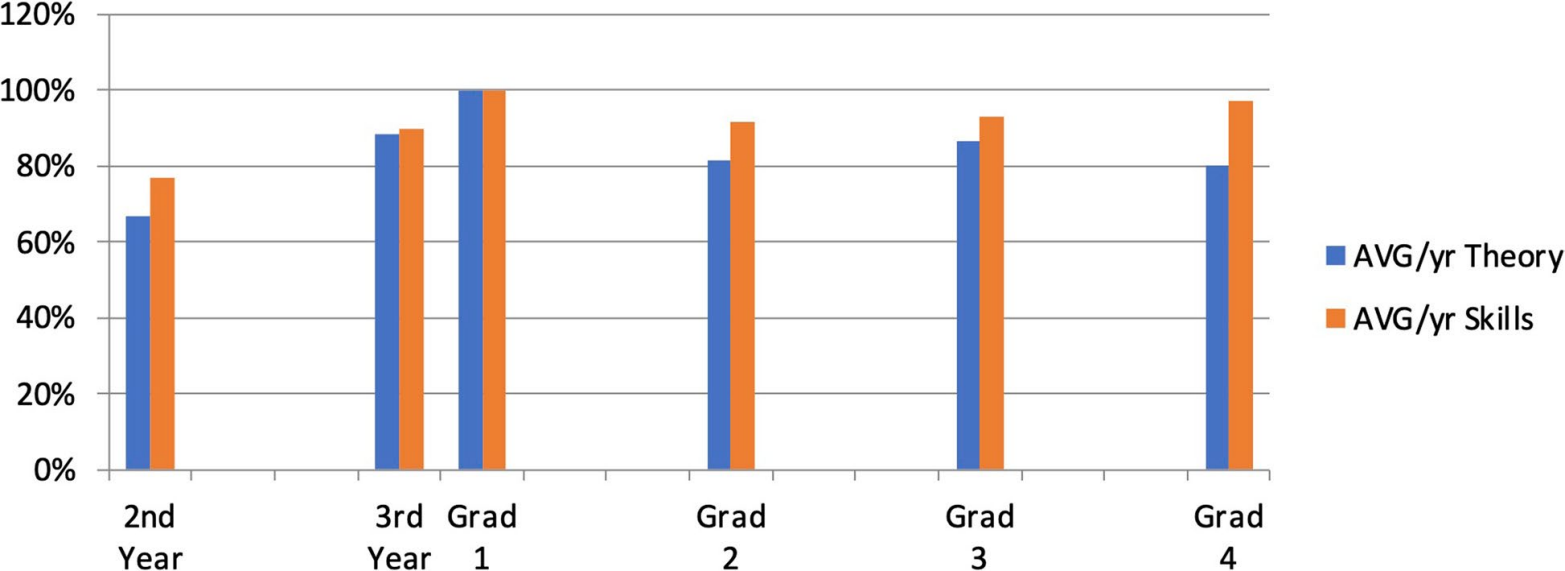
Graph showing the average scores of the ED physicians in the theory and practical aspects of the evaluation

The physicians that had graduated from the residency program were divided into four (4) groups designated as follows: Grad 1–Grad 4 based on years since graduation. The Grad 1 group had just graduated from residency, the Grad 2 group graduated from residency between 1 and 3 years ago, the Grad 3 graduated from residency between 3 and 5 years ago, and the Grad 4 group graduated from residency between 5 and 10 years ago. The Grad 1 group included 1 physician who scored 100% in the evaluation. Grad 2 had a composite average of 87%, scoring 82% on the theory portion and 92% on the practical portion. The Grad 3 group had a composite average of 90%, scoring 87% on the theory portion and 93% on the practical. The Grad 4 group had a composite average of 89% scoring 80% on the theory portion and 97% on the practical portion. Figure 1 displays the composite average scores on the competency assessment for each of the groups. Figure 2 displays the breakdown of the groups’ average scores for the theory and practical portions of the competency assessment.

The majority of physicians had a composite average score above the predetermined pass rate, 80%. All of the residency graduates met the level of competency. The second-year residents were the only physicians that were below the marker of competency.

**Fig. 3:**
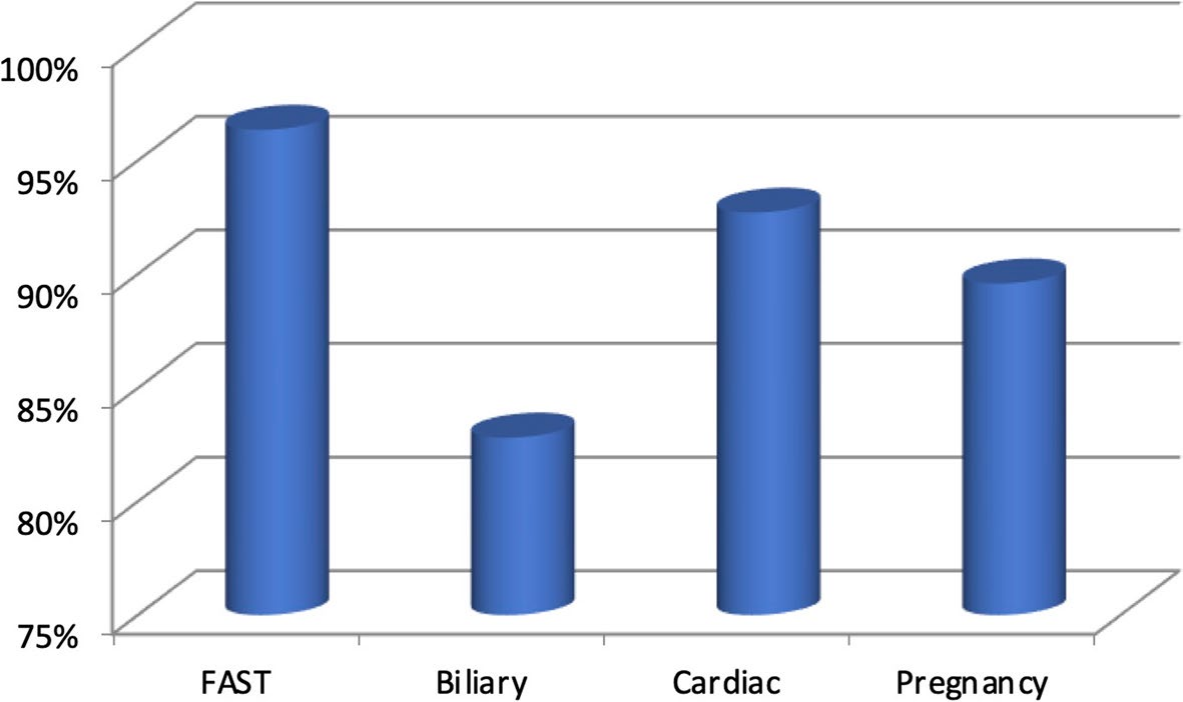
Graph showing the average scores in the four core POCUS examinations evaluated

**Fig. 4:**
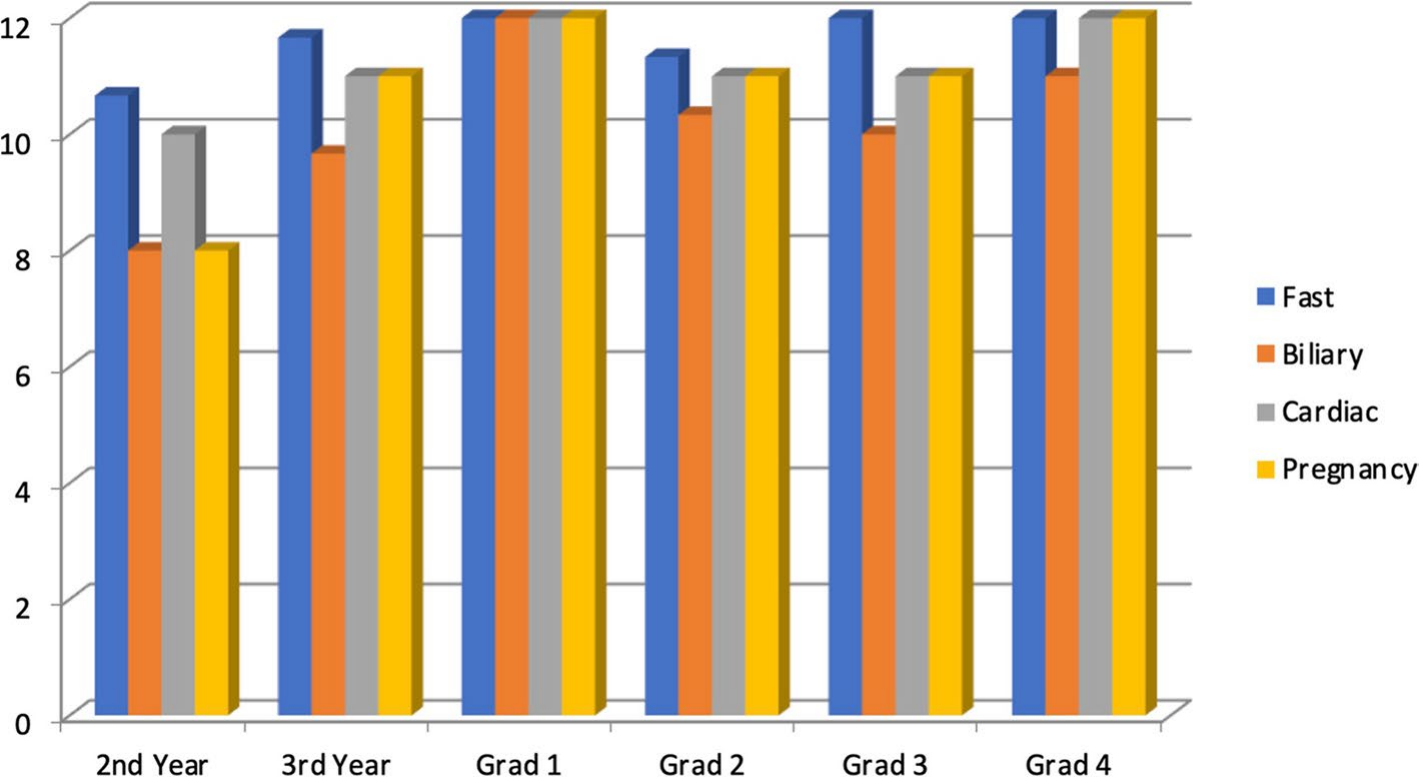
The scores of the different levels of physicians in the core POCUS examinations

Figure 3 displays the assessment results based on individual core POCUS examinations separated into the study groups. The EFAST exam (96%) and cardiac exam (93%) were done with the most proficiency. The biliary exam proved the most difficult with a combined average score of 83% (Fig. 3). Figure 4 displays the evaluation scores based on the individual core applications across each of the different levels of physician experience. All participating physicians scored higher in the practical aspect of the evaluation. There was a correlation between years of experience and performance on the practical portion of the testing. The more experienced providers performed better on the practical portion.

## Discussion

POCUS is a powerful tool for an emergency physician, especially where resources are limited. GPHC has had an established ultrasound curriculum built into residency training since 2016 [4]. While the total sample size is small in this pilot study, this data set represents a majority of the practicing emergency medicine-trained physician in Guyana. All senior residents and residency graduates were competent to acquire and interpret ultrasound images based on this assessment. The data gathered as a part of this study helps identify areas for improvement of the residency curriculum and identifies areas for continuing education for registrars who have previously completed residency training.

There was a positive association with training levels and practical skills of POCUS. The data collected showed that the more experienced graduates had better practical skills, but the theoretical evaluations were more evenly scored. The second-year residents scored the lowest and were the only ones to not pass the evaluation. This was expected given that they are not fully completed training. There was one physician that scored 100% in the evaluation. The physician is the most recent graduate from the emergency medicine residency at GPHC. This could have been because the physician is fully trained in the core competencies of POCUS, and his skills were not diminished due to time as the more experienced physicians.

Physicians were more competent in the more frequently utilized ultrasound applications, specifically EFAST and cardiac. Competency scores were lower for less frequently used and more technically challenging applicationslike biliary.

One benefit of the training curriculum is that it keeps residency graduates engaged in ultrasound image acquisition and interpretation. The required scans and especially the required observed scans necessitate the registrars participate in the process. The clinical necessity of ultrasound in clinical care also keeps residency graduates engaged with ultrasound. These are possible factors contributing to the sustained competency of registrars distant from graduation.

### Limitations

There were various limitations to this study. This was a single-center pilot study with a very small sample size assessing the competency of 16 emergency physicians at POCUS. The sample represents 88.9% of the physicians in Guyana that meet the inclusion criteria, meaning two physicians were not able to participate. The main limitation to participation was physical distance from Georgetown where the assessments occurred. Although the UCAT was used to assess the physicians, there is risk of subjectivity in the assessments. To optimize continuity and objectivity, the assessments were performed by the same individual.

## Conclusion

The assessment results suggest that the current residency ultrasound training curriculum adapted to the resources available is associated with sustained competency after graduation. There was minor attrition of knowledge amongst the senior physicians, but all senior residents and all participating residency graduates were competent in the core ultrasound applications. At GPHC, residency training and continuing medical education for residency graduates will target knowledge gaps identified to help prevent attrition of POCUS knowledge. Similar institutions may use this information to guide their ultrasound continuing education efforts for emergency medicine physicians.

## Data Availability

All data and material are available for review upon request.

## Abbreviations

EFAST: Extended Focused Assessment with Sonography in Trauma
POCUS: Point-of-care ultrasound
ED: Emergency department
GPHC: Georgetown Public Hospital Corporation
GMO: General medical officer
UCAT: Ultrasound Competency Assessment Tool
